# The association between perseverative negative cognitive processes and negative affect in people with long term conditions: a protocol for systematic review and meta-analysis

**DOI:** 10.1186/2046-4053-3-5

**Published:** 2014-01-06

**Authors:** Leanne Trick, Edward Watkins, Chris Dickens

**Affiliations:** 1 University of Exeter Medical School, College House, St Luke’s Campus, Heavitree Road, Exeter EX1 2LU, UK; 2 Mood Disorders Centre, University of Exeter, Perry Road, Exeter EX4 4QG, UK

**Keywords:** Depression, Anxiety, Negative affect, Perseverative negative cognitive processes, Worry, Rumination, Long term conditions, Systematic review, Meta-analysis

## Abstract

**Background:**

Depression is common in people with long term conditions (LTCs) and is associated with worse medical outcomes. Understanding the mechanisms underpinning this relationship could help predict who is at increased risk of adverse medical outcomes, and lead to the development of novel interventions. Perseverative negative cognitive processes, such as worry and rumination, involve repetitive and frequent thoughts about oneself and one’s concerns. These processes have been associated with negative affect, and also adverse medical outcomes. The results of prospective studies, which would allow causal inferences to be drawn, are more equivocal however. Furthermore, the majority of studies have been conducted in physically healthy individuals, and we do not know to what extent these findings will generalise to people with LTCs.

**Methods/design:**

Electronic databases will be searched using a search strategy including controlled vocabulary and text words related to perseverative negative cognitive processes (such as worry and rumination) and negative affect (including depression and anxiety). Records will be hand-searched for terms related to LTCs. Citation and bibliography searching will be conducted, and authors of included studies will be contacted to identify unpublished studies. Studies will be included if they contain a standardised measure of the prospective association between perseverative negative cognitive processes and negative affect, or *vice versa*, in people with LTCs. Narrative and meta-analytic methods will be used to synthesize the data collected.

**Discussion:**

This review will identify and synthesise studies of the prospective association between perseverative negative cognitive processes and negative affect among people with LTCs. The findings will help to identify whether worry and rumination could cause depression and anxiety in people with LTCs, and might indicate whether perseverative negative cognitive processes are appropriate targets for treatment.

## Background

Depression is common in people with chronic physical illnesses (that is, long term conditions, (LTCs)) and is associated with worse medical outcomes, such as increased mortality, increased morbidity, worse health-related quality of life and increased healthcare utilisation. The causes of depression in people with LTCs and the mechanism underpinning the association between depression and poor medical outcomes remain unclear, however. Understanding the causes of depression and the mechanisms by which depression can influence physical health outcomes among people with LTCs could help predict who is at increased risk of adverse medical outcomes, and could lead to the development of novel psychological interventions that have the potential to improve medical outcomes in addition to improving mood.

Perseverative negative cognitive processes, such as worry and rumination, are repetitive, prolonged and recurrent thoughts about oneself and one’s concerns. Perseverative negative cognitive processes have been shown to be associated with negative affect including the onset, maintenance and relapse of depression for example [1-6]. These processes have also been linked to poor cardiovascular health, impaired wound healing and immune dysfunction [7-9]. While cross-sectional studies support the association between perseverative negative cognitive processes and negative affect, the results of prospective studies, which would allow tentative causal inferences to be made, have been more equivocal [10-14]. Furthermore, the majority of studies have been conducted in physically healthy individuals, and we do not know to what extent these findings will generalise to people with LTCs.

The aim of this review is to determine the strength of the prospective association between perseverative negative cognitive processes and subsequent negative affect among people with LTCs. This might indicate whether perseverative negative cognitive processes could cause depression in people with LTCs, thereby justifying the development and evaluation of interventions that target these processes among people with LTCs.

## Methods/design

This systematic review will be conducted following the guidance of the Centre for Reviews and Dissemination (CRD) [15] and will be reported in accordance with the Preferred Reporting Items for Systematic Reviews and Meta-Analyses (PRISMA) statement [16]. This review is not registered with the International Prospective Register of Systematic Reviews (PROSPERO) as it is outside the scope of the register.

### Inclusion and exclusion criteria

To be eligible for inclusion, studies should attempt to address whether, or to what extent, perseverative negative cognitive processes are prospectively associated with negative affect, or *vice versa*, in people with LTCs. In this context, we define perseverative negative cognitive processes as repetitive, prolonged and recurrent thoughts about oneself and one’s concerns (including worry and rumination), and we use negative affect to refer to anxiety, depression, and negative mood. We define LTCs broadly as conditions which cannot currently be cured but which can be managed with treatment [17].

#### Population

Studies in individuals with any LTC will be included. Only studies in adults (> 16 years old) will be included, and there will be no gender restriction.

#### Interventions

As the focus of this review is on the association between perseverative negative cognitive processes and negative affect, the use of an intervention is not a requirement. However, we anticipate that some experimental or quasi-experimental studies will involve the induction of one or more perseverative negative cognitive processes or negative affect whilst observing change in the other domain, and these studies will be included.

#### Comparators

This review will include observational studies, so use of a comparator is not a requirement for inclusion. In the case of experimental or quasi-experimental studies in which perseverative negative cognitive processes or negative affect are induced, we will include studies if the comparator condition enables isolation of the effect of the intervention, for example where a comparator group receives no induction, or a sham induction.

#### Outcomes

In order to be included studies must contain a standardised measure of at least one type of perseverative negative cognitive process (for example, worry or rumination) and a standardised measure of at least one type of negative affect (depression, anxiety, or negative mood). We will include studies if they have measured the variables of interest, and will contact authors for additional details where the outcomes of such measures are not reported. We will extract data on physical outcomes as well as negative affect where such data are presented.

#### Study design

Observational prospective cohort studies, prospective longitudinal studies, and experimental or quasi-experimental studies (before-and-after studies) will be included in this review. Retrospective and cross-sectional studies will not be included.

#### Other limiters

There will be no date or language restrictions. Studies published as papers in peer reviewed journals will be included; studies in reports, book chapters, conference abstracts, or dissertations will be included if they contain sufficient information. Authors will be contacted for additional information if necessary.

### Search strategy

We will search the following electronic databases, from inception, using the same search strategy with alterations as appropriate for each database: MEDLINE, Excerpta Medica DataBase (EMBASE), PsycINFO, and Cumulative Index to Nursing and Allied Health Literature (CINAHL). Search terms will include controlled vocabulary and text-words, and details of the search strategy are provided in an additional file [see Additional file 1]. Records will be hand-searched for terms related to LTCs. We will conduct citation and bibliography searches of included studies to identify further relevant studies, and will attempt to identify unpublished studies by contacting authors of included studies. Electronic database searches will be repeated once the review is complete in order to identify studies that emerge after the initial searches but prior to publication of findings.

### Study selection

One researcher will conduct the electronic database searches and export the records to EndNote X6. Duplicates will be identified and deleted by the same researcher. Eligibility screening will take place in two stages. All titles and abstracts will be independently screened by two reviewers against the inclusion/exclusion criteria to identify potentially relevant studies. Studies that do not meet specific inclusion/exclusion criteria will be rejected at this stage, and the reason for rejection will be recorded. Disagreements between the two reviewers will be resolved by discussion, with the involvement of a third reviewer where agreement cannot be reached. Next, full text copies of all remaining studies will be obtained and independently assessed for inclusion by two reviewers. At this stage, records will be hand-searched to include only populations with LTCs. Again, studies that do not meet specific inclusion/exclusion criteria will be rejected at this stage, and the reason for rejection will be recorded. Disagreements between the two reviewers will be resolved by discussion, with the involvement of a third reviewer where necessary. Multiple reports of the same study will be counted only once; the record containing the greatest amount of information (for example, largest sample size, or longest follow-up period) will be retained. A flow chart showing details of studies included and excluded at each stage of the eligibility screening process will be produced following the PRISMA template.

### Data extraction

Data from all included studies will be extracted independently by two reviewers into a standardised data extraction form which will be piloted on a sample of five studies and then modified if necessary before full data extraction begins. A list of variables to be extracted is shown in an additional file [see Additional file 2]. Discrepancies will be resolved by discussion, with the involvement of a third reviewer where necessary. Authors of included studies will be contacted to provide missing or additional data where necessary. Data extraction will include details of study design; sample characteristics and demographics; measures of perseverative negative cognitive processes and negative affect used and frequency of measurements; measures of physical health/medical outcomes; experimental or quasi-experimental manipulations; statistical methods including variables controlled for in analyses; outcomes of statistical analyses; and information relating to quality assessment.

### Quality assessment

The Effective Public Health Practice Project (EPHPP) Quality Assessment Tool [18] will be used to assess the quality of each study. Ratings will be made for the following components: selection bias, study design, confounders, blinding, data collection methods, withdrawals and drop-outs. Each component will be rated strong, moderate or weak, and these component ratings will also be combined into a global quality rating (strong, moderate or weak). Quality will be assessed by two reviewers, and discrepancies resolved by discussion.

### Data synthesis

Characteristics and findings of included studies will be summarised in tables, and a narrative description will be presented. Studies will be meta-analysed where they contain a statistical estimate of the prospective association between at least one measure of perseverative negative cognitive processes and negative affect, or vice versa, in people with LTCs. Where a statistical estimate is not available, authors will be contacted to provide additional information. Effect sizes will be calculated for each independent study using standardised mean differences or odds ratios, depending on the predominant way results have been presented in included studies. Only one effect per study will be included in the meta-analysis, based on the analysis in which fewest additional variables are controlled for in order to reduce heterogeneity. Where results for a single population are presented in multiple publications, a single effect size only will be calculated. Where studies present effects on the prospective association at multiple follow-up times, a single follow-up period will be chosen for inclusion in the meta-analysis, most probably that closest to the median of the other studies to reduce heterogeneity. Effect sizes will be pooled using random effects models, weighted using the inverse of the variance. Results will be presented in forest plots with the combined effect (95% confidence intervals). Heterogeneity among included studies will be investigated using the I^2^ statistic (where thresholds of < 25% will be taken to suggest low heterogeneity, < 50% to suggest moderate heterogeneity, and > 75% to suggest high heterogeneity, as per [19]) and Cochran’s Q test (where the significance level for Chi squared will be set at *P* = 0.1). If sufficient data are available, variation in effects on the prospective association across characteristics of i) the study population, and ii) the methodology of the study will be calculated using random effects, univariate meta-regression (continuous variables), and the analogue to Analysis of Variance (categorical variables). Publication bias will be assessed using funnel plots (if there are ≥ ten studies) and Egger’s regression method. Sensitivity analyses will be conducted to investigate the influence of study quality on the findings of the meta-analysis; this will be achieved by repeating the meta-analysis with studies of the weakest quality omitted.

## Discussion

This systematic review will identify and synthesise evidence of the prospective association between perseverative negative cognitive processes and negative affect in people with LTCs, to determine the strength of association and the extent to which the effect varies with methodological and population characteristics. The findings of this review will help to identify whether perseverative negative cognitive processes such as worry and rumination could cause depression and anxiety in people with LTCs, and whether these processes are relevant targets for treatment aimed at reducing depression and anxiety in people with LTCs.

## Abbreviations

CINAHL: Cumulative index to nursing and allied health literature; CRD: Centre for reviews and dissemination; EMBASE: Excerpta medica DataBase; EPHPP: Effective public health practice project; LTCs: Long term conditions; PRISMA: Preferred reporting items for systematic reviews and meta-analysis; PROSPERO: International prospective register of systematic reviews.

## Competing interests

The authors declare that they have no competing interests.

## Authors’ contributions

LT, EW and CD participated in the design of the protocol and helped to draft the manuscript. All authors read and approved the final manuscript.

## Authors’ information

LT is a PhD student at the University of Exeter Medical School under the supervision of CD and EW.

EW is Professor of Experimental and Applied Clinical Psychology at the University of Exeter, and Director of the Mood Disorders Centre (University of Exeter/Devon Partnership NHS Trust).

CD is Professor of Psychological Medicine at the Institute of Health Research, University of Exeter Medical School, and Honorary Consultant in Psychological Medicine at the Devon Partnership NHS Trust.

## Supplementary Material

Additional file 1 **Search strategy.** Details of search terms used to build the search strategy and conduct database searches. Click here for file

Additional file 2 **Data extraction list.** List of variables for which information will be extracted from included studies. Click here for file
