# Supplementary material for: The association between perseverative negative cognitive processes and negative affect in people with long term conditions: a protocol for systematic review and meta-analysis
Source: Syst Rev. 2014 Jan 6;3:5. doi: 10.1186/2046-4053-3-5 (PMC3896701; doi:10.1186/2046-4053-3-5)
Supplement: Additional file 1 — Search strategy. Details of search terms used to build the search strategy and conduct database searches. [file 2046-4053-3-5-S1.docx]

**Search strategy**

1 depression.ti,ab,sh.

2 depressive disorder.ti,ab,sh.

3 anxiety.ti,ab,sh.

4 anxiety disorder*.ti,ab. or anxiety disorders.sh.

5 stress, psychological.sh.

6 psychological distress.ti,ab.

7 emotional distress.ti,ab.

8 1 or 2 or 3 or 4 or 5 or 6 or 7

9 perseverative.ti,ab. and cognition.ti,ab,sh.

10 (perseverative and cognitive and processes).ti,ab.

11 perseverative.ti,ab. and thinking.ti,ab,sh.

12 (perseverative and thought).ti,ab.

13 repetitive.ti,ab. and thinking.ti,ab,sh.

14 (repetitive and thought).ti,ab.

15 (worry* or worrie* or worrisome).ti,ab.

16 ruminat*.ti,ab.

17 response styles theory.ti,ab.

18 brooding.ti,ab.

19 preoccupation.ti,ab.

20 (self focus or self focused attention).ti,ab.

21 emotion regulation.ti,ab.

22 coping strateg*.ti,ab.

23 coping style.ti,ab.

24 9 or 10 or 11 or 12 or 13 or 14 or 15 or 16 or 17 or 18 or 19 or 20 or 21 or 22 or 23

25 longitudinal studies.sh. or longitudinal study.ti,ab.

26 prospective studies.sh. or prospective study.ti,ab.

27 followup studies.sh. or follow up.ti,ab.

28 baseline.ti,ab.

29 experience sampling.ti,ab.

30 time series.ti,ab.

31 induction*.ti,ab.

32 25 or 26 or 27 or 28 or 29 or 30 or 31

33 8 and 24 and 32
