# Supplementary material for: The association between perseverative negative cognitive processes and negative affect in people with long term conditions: a protocol for systematic review and meta-analysis
Source: Syst Rev. 2014 Jan 6;3:5. doi: 10.1186/2046-4053-3-5 (PMC3896701; doi:10.1186/2046-4053-3-5)
Supplement: Additional file 2 — Data extraction list. List of variables for which information will be extracted from included studies. [file 2046-4053-3-5-S2.docx]

**Data extraction list**

The following list represents variables for which information will be extracted (where applicable) from included studies into a custom-designed Excel 2010 spreadsheet.

| **Record details** | | | |
| --- | --- | --- | --- |
| Data extractor |  | | |
| Unique study identifier |  | | |
| Endnote accession number |  | | |
| Authors |  | | |
| Year of publication |  | | |
| Title |  | | |
| Citation |  | | |
| Publication type |  | | |
| **Study design** | | | |
| Study design |  | | |
| Number of follow-up assessments |  | | |
| Timing of follow-up assessments |  | | |
| Experimental intervention/manipulation* |  | | |
| Control condition* |  | | |
| Study aim / objective |  | | |
| Other notes |  | | |
| **Sample characteristics** | | | |
| Details of long term condition |  | | |
| Mean age |  | | |
| Proportion males |  | | |
| Ethnicity |  | | |
| Recruited from |  | | |
| Sampling strategy |  | | |
| Country |  | | |
| Sample size: recruited |  | | |
| Sample size: analysed |  | | |
| Physical health |  | | |
| Mental health |  | | |
| Other notes |  | | |
| **Measures** | | | |
| Measure/s of PNCP^#^ (description plus frequency and timing of assessments) | |  | |
| Measure/s depression (description plus frequency and timing of assessments) | |  | |
| Measure/s of anxiety (description plus frequency and timing of assessments) | |  | |
| Measure/s of negative mood (description plus frequency and timing of assessments) | |  | |
| Physical outcome measures | |  | |
| Other notes | |  | |
| **Outcomes** | | | |
| **Bivariate analysis** |  | | |
| Description of bivariate analysis |  | | |
| Measure of PNCP^#^ |  | | |
| Measure of negative affect |  | | |
| Outcome – test statistic (e.g. r) |  | | |
| Outcome – probability value (p =) |  | | |
| **Multivariate analysis** |  | | |
| Description of multivariate analysis |  | | |
| Measure/s of PNCP^#^ |  | | |
| Measure/s of negative affect |  | | |
| Independent variable (predictor/s) |  | | |
| Dependent variable (outcome/s) |  | | |
| Variables controlled for |  | | |
| Outcome – test statistic (e.g. β) |  | | |
| Outcome – probability value (p=) |  | | |
| Total variance accounted for by model |  | | |
| **Other** |  | | |
| Other analyses – description and outcomes |  | | |
| Other notes |  | | |
| **Quality assessment – EPHPP criteria** | | | |
| Selection bias | | |  |
| Study design | | |  |
| Confounders | | |  |
| Blinding | | |  |
| Data collection methods | | |  |
| Withdrawals and drop-outs | | |  |
| Intervention integrity | | |  |
| Analyses | | |  |
| Other notes | | |  |

* Applicable to experimental / quasi-experimental / before-and-after studies but not to longitudinal cohort studies involving no intervention / manipulation

^#^ Perseverative negative cognitive processes
